# Cortical indices, DXA, and FRAX as indicators of bone fragility: a comparative human cadaveric study

**DOI:** 10.1093/jbmrpl/ziag155

**Published:** 2026-09-14

**Authors:** Anthony J Yosick, Sophia L Turbide, Andrew J Berger, Hani A Awad

**Affiliations:** Department of Biomedical Engineering, University of Rochester, Rochester, NY 14627, United States; Center for Musculoskeletal Research, Department of Orthopaedics, University of Rochester Medical Center, Rochester, NY 14642, United States; Department of Internal Medicine and Pediatrics, University at Buffalo, Buffalo, NY 14203, United States; Department of Biomedical Engineering, University of Rochester, Rochester, NY 14627, United States; The Institute of Optics, University of Rochester, Rochester, NY 14627, United States; Department of Biomedical Engineering, University of Rochester, Rochester, NY 14627, United States; Center for Musculoskeletal Research, Department of Orthopaedics, University of Rochester Medical Center, Rochester, NY 14642, United States; Department of Orthopaedics, University of Rochester Medical Center, Rochester, NY 14642, United States

**Keywords:** osteoporosis, fracture, fracture risk, screening, FRAX, DXA, cortical index

## Abstract

Osteoporosis (OP) is a progressive skeletal disorder characterized by reduced BMD and increased risk of fragility fractures. Clinical assessment relies primarily on DXA and the fracture risk assessment tool (FRAX), but limited access and low screening rates motivate investigation of complementary approaches. This human cadaveric study evaluated cortical indices of the second metacarpal, radius, and femur in relation to DXA-based OP status and biomechanically derived fracture susceptibility at the wrist and hip. Cortical measurements were obtained from 39 donor-matched forearms and femurs categorized as normal (*n* = 8), osteopenic (*n* = 11), and osteoporotic (*n* = 20) based on femoral neck (FN) DXA T-score. Simulated fall testing of the distal radius and proximal femur was performed to determine work to fracture, which was used to derive low and high biomechanical fracture susceptibility classifications. Among the cortical indices, metacarpal cortical index (MCI) demonstrated the strongest associations with DXA T-scores and biomechanical outcomes. MCI distinguished normal from osteoporotic classification at the FN (area under the curve [AUC] = 0.938) and 1/3 radius (AUC = 0.986). For biomechanically derived fracture susceptibility, FN T-score demonstrated strong discrimination at both the femur (AUC = 0.917) and wrist (AUC = 0.851), while MCI yielded AUCs of 0.847 and 0.855, respectively. FRAX hip fracture probability yielded AUCs of 0.911 at the femur and 0.830 at the wrist. These findings demonstrate associations between MCI, DXA-based OP status, and biomechanical fracture susceptibility in this cadaveric cohort. MCI may therefore warrant further investigation as a complementary screening approach, particularly where access to DXA is limited. Validation in larger clinical cohorts with prospective fracture outcomes is needed to determine its clinical utility.

## Introduction

Osteoporosis (OP) is a silent bone disease. Without effective screening, diagnosis, and treatment, bone mineral density (BMD) loss often goes unnoticed until a fragility fracture occurs. Annually, it is estimated that 37 million fragility fractures occur in individuals over 55 years of age.^1^ While OP is indiscriminate of gender, it predominately impacts women with 1 in 3 over 50 years of age experiencing a fracture, nearly double the rates in men.^2^ Most commonly, fragility fractures manifest at the proximal humerus, distal forearm (wrist), proximal femur (hip), or vertebrae (spine).^3^ Among women at 45 years of age, the distal forearm and proximal femur have the highest remaining lifetime risk of fracture at 21.5% and 23.3%, respectively.^4^ Fragility fractures represent a leading medical expense among women in the United States, with estimated annual costs exceeding $22 billion, surpassing those of breast cancer, myocardial infarction, and stroke, while also contributing significantly to both increased morbidity and mortality.^5^ Hip fractures (HFs) are the most severe, often requiring hospitalization. Nearly 25% of patients die within 1 year of a HF, and of those who survive, approximately 30% lose their independence. Identification of OP at the time of an initial fracture, followed by appropriate treatment, can reduce the risk of subsequent fractures by nearly 50%.^3^ This underscores the critical need for effective and accessible screening methods for fracture risk identification where standard screening tools are otherwise unavailable.

Osteoporosis is typically evaluated by measuring BMD through DXA. BMD serves as the basis for calculating a T-score, the number of standard deviations from a normal, healthy BMD of the same sex, and ethnicity. Osteoporosis is defined by the diagnostic threshold of 2.5 standard deviations below this reference (T-score ≤ −2.5). Clinically, DXA is commonly acquired at the lumbar spine, hip (femoral neck), or the forearm (radius).^6^^,^^7^ Although effective at quantitatively characterizing bone loss, DXA T-score has some limitations in accurately describing fracture risk, prompting development of more health history centric tools such as the fracture risk assessment tool (FRAX). Fracture risk assessment tool expands upon FN BMD by incorporating additional clinical risk factors including age, height, prior fractures, smoking, excessive alcohol use, glucocorticoid use, rheumatoid arthritis, and secondary OP. These risk factors are used to estimate a 10-year probability of fracture categorized as either a major osteoporotic fracture (MOF; including fractures of the wrist, hip, humerus, or spine) or HF.

Although DXA T-score is established as the World Health Organization’s (WHO) clinical gold-standard for OP diagnosis and monitoring, its utility is undermined by alarmingly low screening rates in clinical practice, leaving many at-risk individuals undiagnosed and untreated. In a study conducted from 2008 to 2014, DXA screening rates for initial and recurring scans of women aged 50-64, 65-79, and 80+ years of age were 21.1%, 26.5%, and 12.8%, respectively.^8^ In a study commissioned by the National Osteoporosis Foundation, it was found that up to 91% of women over 65 years of age with a prior low-trauma fracture had not been screened for OP.^9^ The underutilization of DXA screening across this highest risk population is apparent. Factors driving these dismal rates range from changes in reimbursement rates for scans to availability and access to DXA facilities, particularly in rural settings.^10^^,^^11^ Beyond screening, this directly impacts the value of FRAX assessment, as accurate fracture risk calculation requires DXA BMD. Consequently, there is a clear need for additional accessible screening methods to bridge this gap.

A variety of alternative methods focusing on peripheral bones have been explored as alternatives to the gold-standard tools of DXA and FRAX, including digital X-ray radiogrammetry (DXR), which estimates DXR-BMD by analyzing the cortical thickness, width, and porosity of the metacarpals.^12^ Prospective studies established that DXR predicts wrist, hip, and vertebral fractures as effectively as other peripheral methods, offering an alternative where central DXA is unavailable.^13^^,^^14^ Later research confirmed its ability to predict HF risk in both sexes using standard hand radiographs and demonstrated superior prediction of FN OP compared to FRAX.^15^ Despite this, widespread adoption has been hindered by the accepted status of DXA as the clinical gold standard and the weaker prediction of specific FN fractures by DXR compared to hip DXA. Nevertheless, assessments of OP fracture risk based on digital bone size and geometry, microarchitecture, and composition continues to be an active area of research to opportunistically identify individuals at high risk of OP fractures within standard clinical workflows. In particular, the metacarpal cortical index (MCI) offers a practical and accessible option by utilizing standard hand radiographs to quantify the normalized cortical thickness of the second metacarpal. MCI has demonstrated effectiveness in screening for osteopenia (OPE), guiding decisions around OP screening and antiresorptive therapy, and remotely predicting bone strength and fragility fractures.^16–21^

Despite the promising correlations observed with MCI, several gaps remain in translating these findings to clinical practice. These include the reliance on retrospective fracture analyses, biomechanical testing that may not accurately represent typical fragility fracture loading conditions, and an incomplete understanding of how cortical indices measured at the second metacarpal relate to those at other remote skeletal sites for fragility fracture, the wrist and femur. This study aims to investigate MCI of the second metacarpal in relation to the radial cortical index (RCI) and femoral cortical index (FCI) to evaluate correlations across remote skeletal sites. We then examine whether the cortical index with the strongest correlation can be used to predict DXA T-scores at the 1/3 radius and the FN. Finally, we assess the ability of these cortical indices to predict fracture susceptibility at the wrist and the femur through simulated fragility fracture biomechanical testing and compare it to DXA and FRAX predictions.

## Materials and methods

### Human cadaveric donor population

Human cadaveric specimens were acquired through Anatomy Gifts Registry. This cohort was comprised of 39 fresh-frozen intact cadaver forearms (disarticulated at the elbow) and excised femurs. No matched limbs (left and right forearms or femurs from the same donor) were included. Considering that biological female sex is a known risk factor for OP, the female-only study cohort was chosen to enhance the statistical significance and specificity of the findings. Donor health histories were screened prior to acceptance to ensure all inclusion and exclusion criteria were met. Inclusion criteria for this population included HIV-negative serology, recovery from donor in less than 3-years postmortem, and availability of relevant medical history to enable FRAX estimation. Exclusion criteria included previous fractures on the identified forearm or femur, donors who were non-ambulatory for more than 1-year antemortem, donors with musculoskeletal impairments (paralysis or paresis), presence or history of neoplasm on the identified forearm or femur, and prosthetic hardware on or interfacing with the identified forearm or femur.

### DXA

To categorize donors by WHO classification based upon T-scores, FN DXA scans were acquired ex vivo upon delivery at the University of Rochester Medical Center’s Center for Musculoskeletal Research using a preclinical Insight Vet DXA (OsteoSys Co., Ltd.). The DXA scanner was calibrated prior to each use using a phantom, with measurements required to fall within ±0.003 g/cm^2^ of the target value. All FN DXA scans were acquired in triplicate and an average taken of BMD values. Dissected femoral specimens were position within the DXA scanner with the posterior surface down and anterior surface facing upward. The femur was oriented such that the coronal plane was approximately parallel to the scanner bed. FN T-score was calculated using reference BMD mean and SD values established in Xue et al.^22^ Distal radius DXA scans were collected by Advanced Radiology using a Horizon Ci DXA System (Hologic). 1/3 radius DXA scans were obtained in situ from cadaveric forearm specimens following disarticulation at the elbow.

Throughout this manuscript, DXA WHO classifications are based upon the FN T-scores. Results for 1/3 radius T-scores are presented in selected figures and supplementary materials. A summary of cohort demographics based on FN WHO classification is presented in Table 1. Of the 39 donors, *n* = 8 were normal (*N*), *n* = 11 were OPE, and *n* = 20 were OP. DXA T-score classifications between the 1/3 radius and FN regions resulted in similar levels of agreement with large cohort studies conducted by Moayyeri et al. illustrated in Table S1.^23^ A summary of cohort demographics based on 1/3 radius BMD and T-scores is presented in Table S2.

**Table 1:** Cohort demographics by FN DXA T-score WHO classification.

**Femoral neck T-score classification**						
**WHO classification**	**Donors**	**Ethnicity**	**Age (years)**	**BMI (kg/m** ^**2**^ **)**	**BMD (g/cm** ^**2**^ **)**	**T-score**
**Normal**	*n* = 8	Black: *n* = 1 White: *n* = 7	53.9 ± 8.4	33.8 ± 5.7	0.962 ± 0.134	0.53 ± 1.12
**Osteopenia**	*n* = 11	Black: *n* = 2 White: *n* = 9	66.5 ± 14.6	31.6 ± 9.7	0.683 ± 0.062	−1.76 ± 0.54
**Osteoporosis**	*n* = 20	White: *n* = 20	75.9 ± 8.7	24.0 ± 6.9	0.475 ± 0.074	−3.41 ± 0.61

Demographics are presented as the sample size per classification or as a mean ± standard deviation. Abbreviation: WHO, World Health Organization.

### Fracture risk assessment

Donor health histories provided through Anatomy Gifts Registry were comprehensive from available medical records at the time of death. These histories were reviewed for all variables relevant to the FRAX calculation of MOF and HF. Guidance was established to provide consistency in health history interpretation for current smoking status, alcohol consumption (3 or more units per day), and secondary OP inputs. Donors who smoked within 14-years of death were indicated as “Yes” for current smoking. Studies have indicated that smoking contributes to OP progression up to 14-years following cessation.^24^ Alcohol consumption was generally characterized descriptively in donor health histories. Social, occasional, and mild alcohol consumption were indicated as “No” for alcohol consumption whereas moderate was indicated as “Yes” for alcohol consumption. Secondary OP was indicated as “Yes” if type I diabetes was listed. FRAX assessments were available for 37 donors, as 2 exceeded the FRAX upper age limit of 90 years. FN BMD was used for all FRAX calculations.

### Cortical index measurements

Cortical indices were measured for second metacarpal, radius, and femoral radiographs. Hand and forearm radiographs were collected using a Hologic UltraFocus Faxitron X-ray system (Hologic, Inc.) and femoral radiographs using an Insight Vet DXA (OsteoSys Co., Ltd.). Representative radiographic images for MCI, RCI, and FCI with equations are illustrated in Figure 1A–C. MCI measurements were collected at the mid-diaphysis of the second metacarpal (Figure 1A). RCI measurements were adapted from radius cortical thickness measurements from Schmidutz et al.^25^ Measurements were collected 2 cm proximal to the point where the outer medial and lateral cortical edges are parallel (Figure 1B). FCI measurements were adapted from cortical thickness index measurements from Nguyen et al.^26^ Measurements were collected 10 cm distal from the mid lesser trochanter (Figure 1C). Cortical index measurements were collected using FIJI (ImageJ2 version 2.16.0/1.54p, build 26d66057dd; Schindelin et al., 2012).^27^

**Figure 1:**
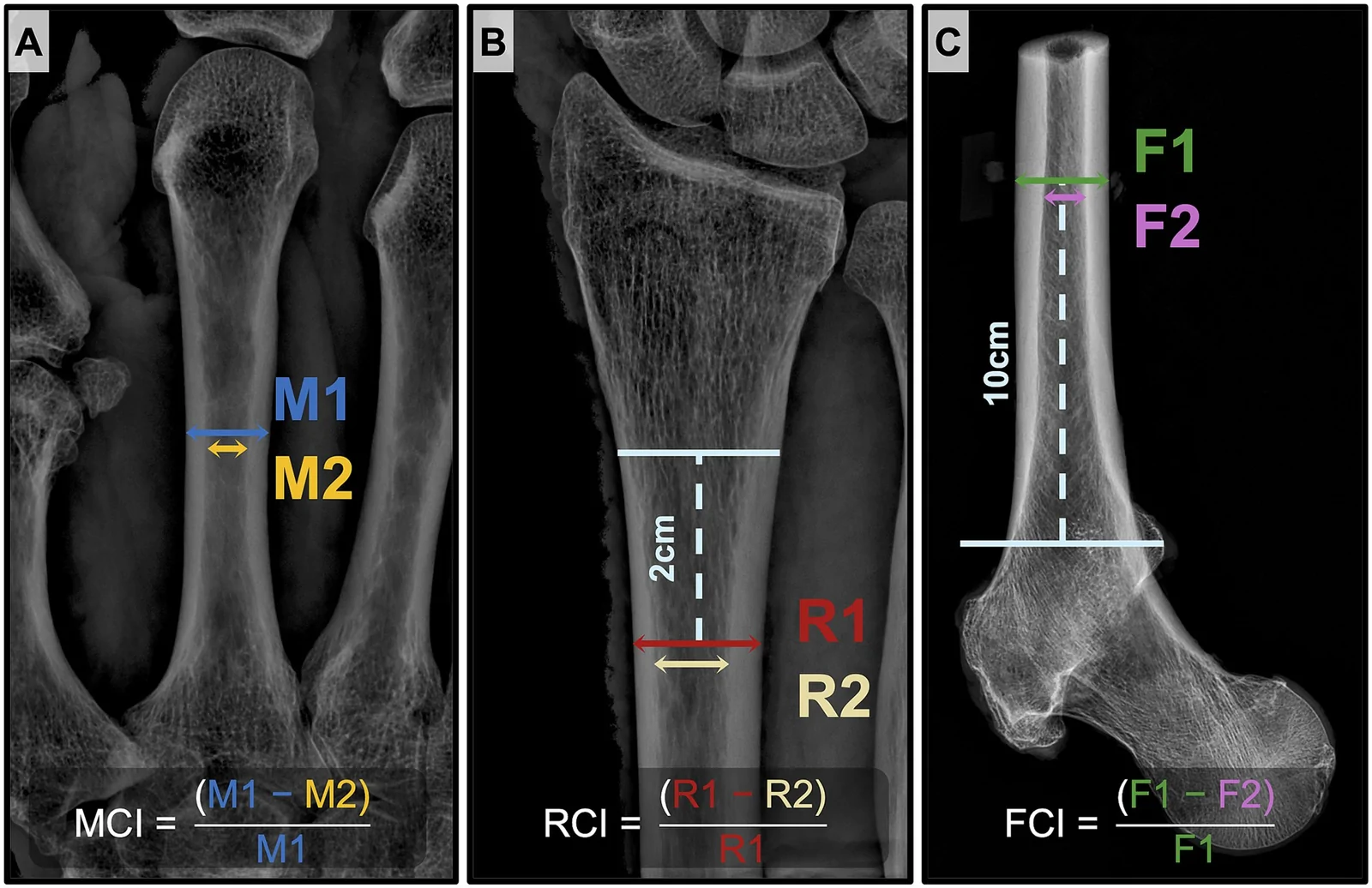
Representative radiographic dimensional analysis of the second metacarpal, radial, and femoral cortical index. (A) MCI at the mid-shaft. (B) RCI at 2 cm proximal to the point where the outer medial and lateral cortical edges are parallel. (C) FCI at 10 cm distal from the mid lesser trochanter. FCI, femoral cortical index; MCI, metacarpal cortical index; RCI, radial cortical index.

### Biomechanical testing

Biomechanical testing simulated common fragility fracture loading for the forearm and femur. Forearm specimens simulated a fall on an outstretched hand (FOOSH). Specimens were stored at −80 °C and thawed for 18-20 h at room temperature prior to dissection. Forearms were dissected approximately 15.2 cm proximal from the ulnar styloid process. Soft tissue was then removed from the proximal portion of the specimen exposing approximately 7.6 cm of the radius and ulna. The specimen was embedded in self-curing acrylic (Bosworth Fastray, Keystone Industries) in pronation and 15° radial abduction (Figure 2A and B).^28–30^

**Figure 2:**
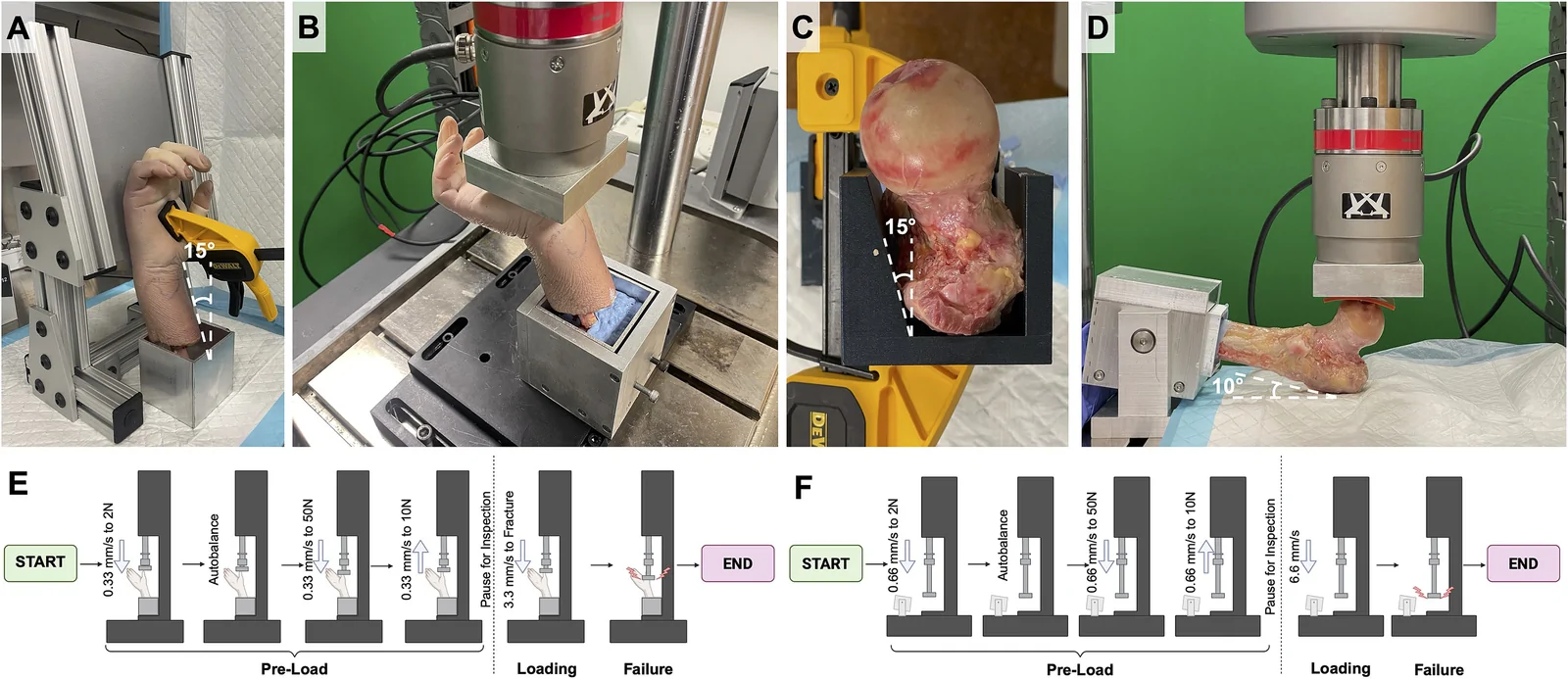
Biomechanical testing setup and protocol for FOOSH and sideways fall on hip. (A and B) FOOSH forearm mounting setup. (C and D) Fall on hip femoral mounting setup. (E) FOOSH biomechanical testing protocol. (F) Fall on hip biomechanical testing protocol. FOOSH, fall on an outstretched hand.

Femoral specimens simulated a sideways fall onto the hip. These specimens were stored at −80 °C and thawed and rehydrated for 2 h in 1× PBS prior to mounting. The exposed femur was dissected approximately 21.6 cm distal from the proximal-most point of the femur and embedded in self-curing acrylic with an internal rotation of 15° from the vertical axis and then mounted with the femoral shaft angled 10° above horizontal (Figure 2C and D).^31–33^

Forearm and femoral specimens were both tested on an ElectroPuls E10000 testing system (Instron) equipped with a 10 kN load cell controlled by Console software (version 10.5) and Bluehill Universal software (version 4.42.31223). A loading platen approximately 7.6 × 7.6 × 2.5 cm either pressed onto the breadth of the palm using the distal palmar crease for alignment or the femoral head. For femoral testing, an approximately 5 × 5×0.3 cm section of 60A silicone was placed underneath the greater trochanter and on the femoral head to reduce localized crushing of bone. Test methods for both models were similar. A pre-load was applied at 1/10th testing rate (0.33 mm/s for forearm and 0.66 mm/s for femur) until the system was autobalanced at 2 N. The system then continued at the same rate compressing to 50 N before releasing to 10 N of applied force. The system then paused to ensure specimen or fixture had not shifted. The specimen was then tested in compression until failure at 3.3 mm/s for forearms and 6.6 mm/s for femurs (Figure 2E and F). Biomechanical parameters including stiffness, maximum force, work-to-maximum force, fracture force, and work-to-fracture force were computed from the force–displacement curves (summarized in Table S3). Work to fracture was used in subsequent correlations because it could be directly compared with published fall-related energy thresholds, allowing classification of each specimen into low or high fracture susceptibility based on clinically relevant impact energies at the wrist and femur.

### Fracture susceptibility classifications

To enhance the clinical relevance and interpretability of the biomechanical results, work-to-fracture values were categorized as indicating either low or high fracture susceptibility based on estimated energy experienced at the wrist or femur during a fall from standing height. A threshold for low (≥19 J) vs high (<19 J) wrist fracture susceptibility was established based on a simulated FOOSH model for an average height and weight female.^34^ Kinematic equations, along with average height for females aged 65 and older and average weight for females aged 60 and older, were used to estimate an energy threshold for femur fracture as low (≥29.94 J) or high (<29.94 J).^35–37^

### Statistical analysis

Fracture susceptibility groups were compared using Welch’s *t-*test. Normality of work-to-fracture values across WHO classifications was assessed using the Shapiro–Wilk test. Homogeneity of variance across groups was assessed using the Brown–Forsythe test. Group differences were evaluated using Welch’s one-way ANOVA followed by Dunnett’s T3 multiple comparison test. These analyses were performed using GraphPad Prism version 10.4.1 (GraphPad Software). FRAX predictor screening for work to fracture through linear regression was performed using JMP Student Edition, version 18.2.2 (SAS Institute Inc.). Optimal cutoffs for T-score, FRAX, and MCI were determined using receiver operating characteristic (ROC) curves and the area under the curve (AUC). Simple linear regression analysis graphs to evaluate associations of T-score, cortical indices, and work to fracture were performed. For each model, the coefficient of determination (*R*^2^), root mean square error (RMSE), and *p*-value associated with the regression slope were calculated. ROC AUC, optimal cutoffs, and linear regression analyses were performed using R (version 4.4.0) within the RStudio environment (version 2024.04.1+748; Posit Software).

## Results

Work to fracture at the wrist and femur was evaluated across bone density classifications. Work-to-fracture values for both the wrist and femur are shown in relation to FN T-score (Figure 3A). Both wrist and femur work-to-fracture values were significantly associated with FN T-score (*p* = .0002 and *p* < .0001, respectively). Whereas work to fracture in the FOOSH biomechanical models followed the expected trend across classifications without reaching statistical significance (Figure 3B), fall-on-hip showed significantly lower work to fracture in the OPE and OP groups compared with the N group (*p* = .0330 and *p* = .0039, respectively; Figure 3C). The remaining comparisons, including N vs OPE and *N* vs OP for the wrist and OPE vs OP for both, followed the expected trends but did not reach statistical significance. Additionally, significant differences were identified when donors were classified according to 1/3 radius T-score classification (Figure S1). For wrist work to fracture, a significant difference was shown between the N and OP groups (*p* = .0332; Figure S1A). For femur work to fracture, a significant difference was observed between N and OP (*p* = .0015; Figure S1B). The relationship between wrist and femur work-to-fracture values were significantly associated (*p* = .0200; Figure 3D). Finally, when work to fracture was categorized into low or high biomechanical fracture susceptibility groups, both wrist and femur models demonstrated significantly lower work to fracture in the high-susceptibility group compared with the low-susceptibility group (*p* = .0002 for the wrist; Figure 3E and *p* < .0001 for the femur; Figure 3F).

**Figure 3:**
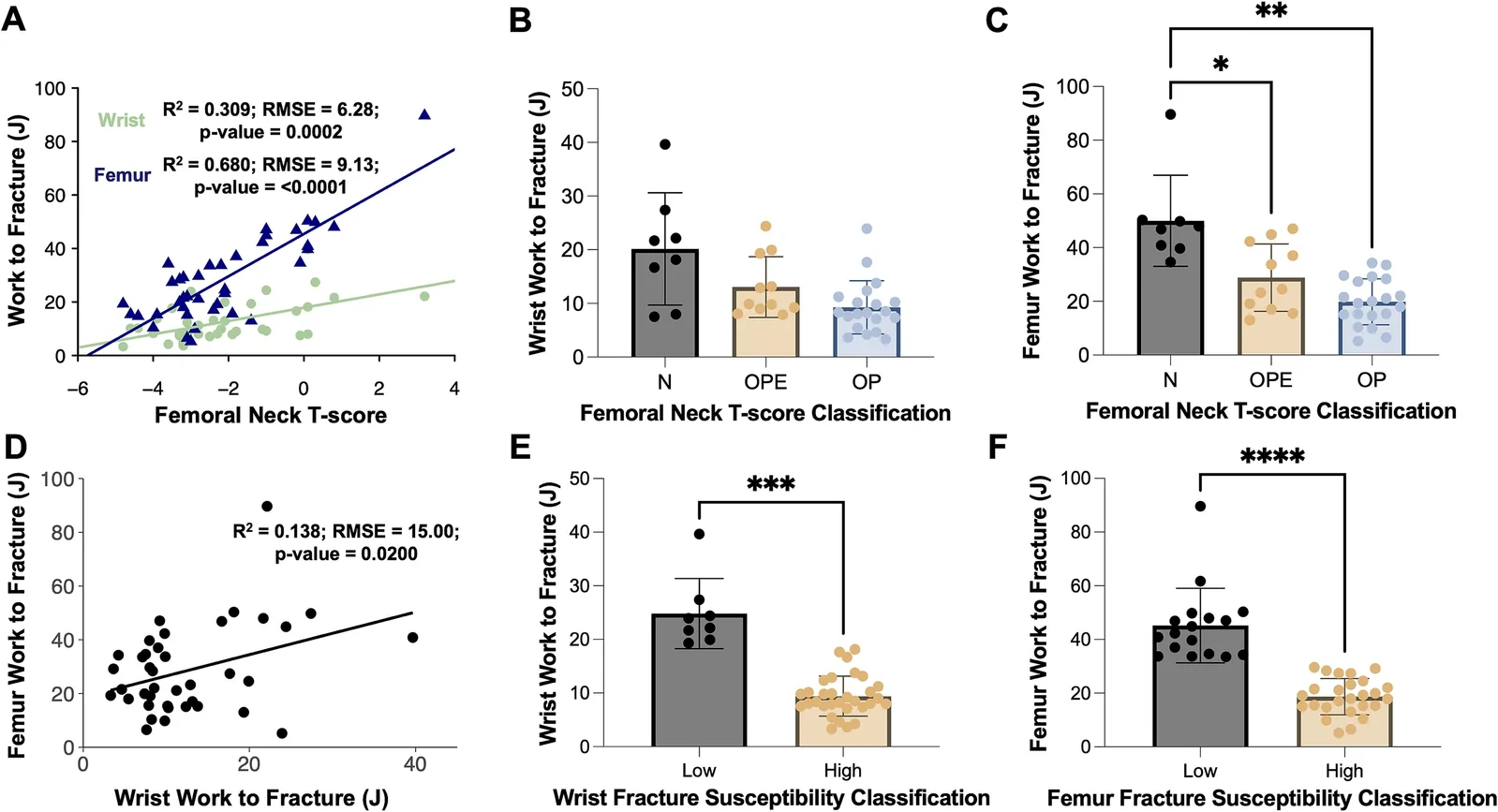
Wrist and femur work-to-fracture values from FOOSH and fall on hip biomechanical models. (A) Wrist and femur work-to-fracture values against FN T-score. Wrist work to fracture categorized by (B) FN DXA T-score classifications and (E) wrist fracture susceptibility classification. Femur work to fracture categorized by (C) FN DXA T-score classifications and (F) femur fracture susceptibility classification. (D) Wrist and femur work-to-fracture values against each other. Asterisks indicate significant differences (^*^p < .05, ^**^p < .01, ^***^p < .001, ^****^p < .0001). FOOSH, fall on an outstretched hand.

Cortical indices were measured at the second metacarpal, radius, and femur across all donors. Mean values and standard deviations for each of these skeletal locations by FN WHO classification are summarized in Table 2. Pairwise comparisons among cortical indices were performed using linear regression. For each comparison, *R*^2^, RMSE, and *p*-value from the regression F-test were calculated and significance was assessed using a Bonferroni correction across the 3 comparisons (Table S4). Among these analyses, the strongest correlations were observed in comparisons involving MCI, particularly for MCI vs FCI (*R*^2^ = 0.246, RMSE = 0.11, *p* = .0007). Each cortical index variable was then individually regressed against FN and 1/3 radius T-score to identify the best-performing predictors (Table S5). MCI demonstrated the highest correlation for both skeletal sites, with *R*^2^ = 0.532, RMSE = 1.29, *p* < .0001 for the 1/3 radius and *R*^2^ = 0.487, RMSE = 1.20, *p* < .0001 for the FN T-score, outperforming both RCI and FCI in each case.

**Table 2:** Cortical indices classified according to FN WHO classification.

**Femoral neck WHO classification**	**MCI**	**RCI**	**FCI**
**Normal**	0.590 ± 0.114	0.282 ± 0.051	0.606 ± 0.048
**Osteopenia**	0.471 ± 0.091	0.274 ± 0.060	0.579 ± 0.053
**Osteoporosis**	0.367 ± 0.085	0.269 ± 0.077	0.511 ± 0.101

Data presented as mean ± standard deviation. Abbreviations: FCI, femoral cortical index; MCI, metacarpal cortical index; RCI, radial cortical index.

ROC analyses were then performed to assess the discriminative ability of the MCI, to classify OP status. AUC values and optimal cutoffs points were calculated to differentiate between N vs OPE, OPE vs OP, and N vs OP across both FN and 1/3 radius (Figure 4). MCI differentiated between OPE and OP (AUC = 0.814, cutoff = 0.426) and between N and OP (AUC = 0.938, cutoff = 0.440; Figure 4A) based on FN T-scores. MCI demonstrated excellent discrimination between OPE and OP (AUC = 0.940, cutoff = 0.409) and between N and OP (AUC = 0.986, cutoff = 0.395; Figure 4B) based on 1/3 radius T-scores. For both classification sites, the categorization between N and OPE yielded AUC values ≥0.78.

**Figure 4:**
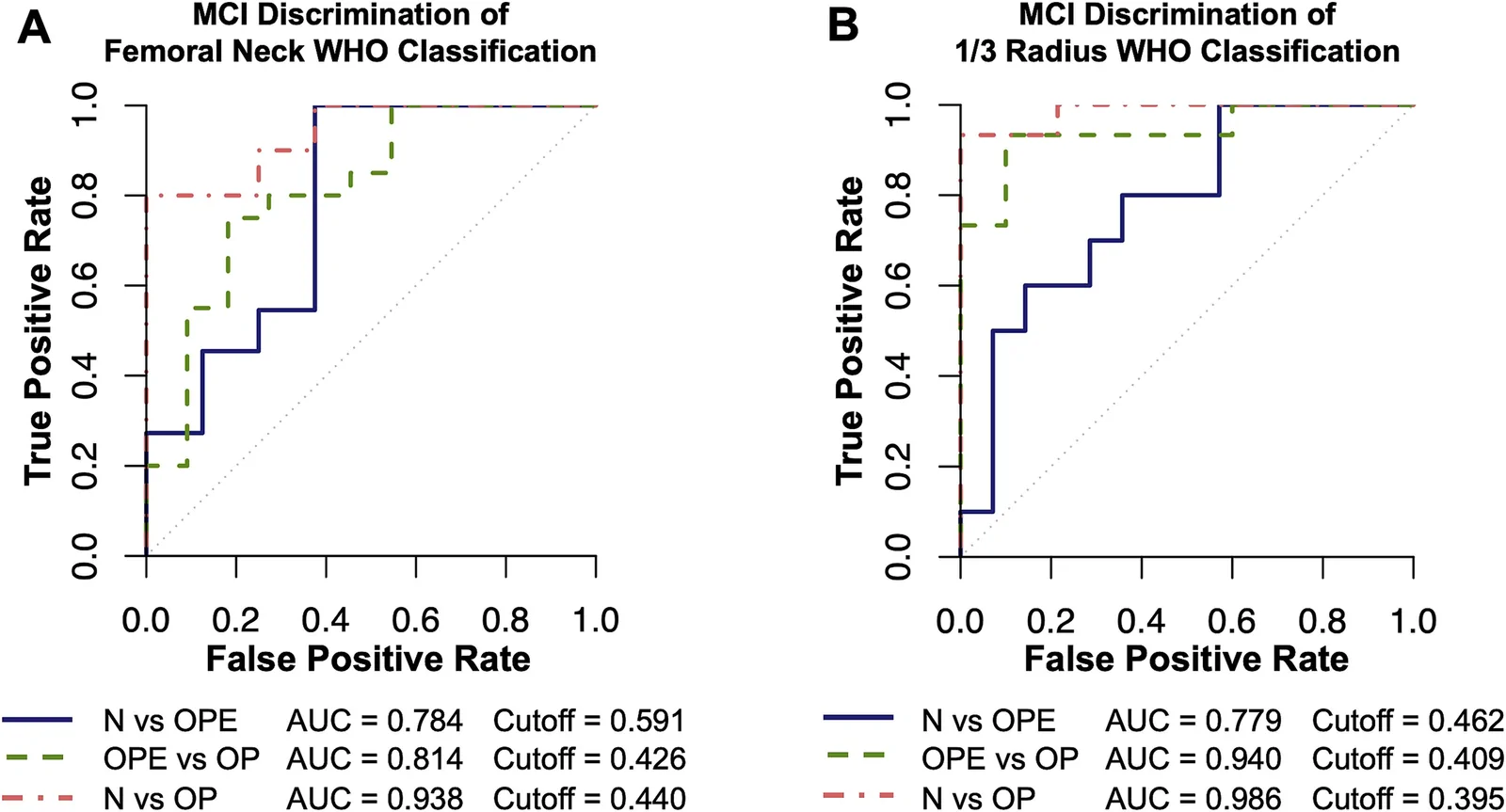
Diagnostic performance of MCI in classifying donors according to FN and 1/3 radius WHO classification. (A) ROC analysis of MCI for predicting the FN DXA T-score categories. (B) ROC analysis of MCI for predicting 1/3 radius DXA T-score categories using the same group comparisons. Curves correspond to N vs OPE, OPE vs OP, and N vs OP classifications. AUC values and optimal cutoff points derived from Youden’s index are shown for each curve, summarizing the discriminative performance of MCI across clinically relevant bone density thresholds. AUC, area under the curve; MCI, metacarpal cortical index; N, normal; OPE, osteopenia; OP, osteoporosis; ROC, receiver operating characteristic.

Simple linear regression was used to evaluate associations between femur and wrist work to fracture and each bone metrics, including cortical indices (MCI, RCI, and FCI), DXA T-scores at the FN and 1/3 radius, and FRAX probabilities of MOF and HF, respectively (Table S6). Among these, the FN T-score showed the strongest performance for predicting both femur (*R*^2^ = 0.680, RMSE = 9.13, *p* < .0001) and wrist (*R*^2^ = 0.309, RMSE = 6.28, *p* = .0002) work to fracture. For wrist work to fracture, MCI (*R*^2^ = 0.206, RMSE = 6.73, *p* = .0037) and the 1/3 radius T-score (*R*^2^ = 0.219, RMSE = 6.85, *p* = .0027) showed comparable predictive performance. However, for femur work to fracture, MCI (*R*^2^ = 0.354, RMSE = 12.98, *p* = <.0001) outperformed the 1/3 radius T-score (*R*^2^ = 0.236, RMSE = 14.57, *p* = .0010). In both analyses, MCI also outperformed the FRAX variables. Individual plots of cortical indices against femur and wrist work-to-fracture values are presented along with *R*^2^, RMSE, and *p*-value associated with the regression slope (Figure S2).

ROC curves were then used to classify wrist and femur work to fracture into low or high biomechanical fracture susceptibility categories based on different predictor values. Overall, FN T-score performed best for both femur (AUC = 0.917; cutoff = −1.8; Figure 5A) and wrist fracture susceptibility (AUC = 0.851; cutoff = −2.1; Figure 5B). FRAX HF performed comparably to FN T-score for classification of femur (AUC = 0.911, cutoff = 5.5%; Figure 5A) and wrist susceptibility (AUC = 0.830, cutoff = 1.4%; Figure 5B). Generally, MCI, 1/3 radius T-score, and FRAX MOF performed comparably. Compared to these clinical standard measurements, MCI effectively predicted susceptibility at both the femur (AUC = 0.847, cutoff = 0.440; Figure 5A) and wrist (AUC = 0.855, cutoff = 0.524; Figure 5B).

**Figure 5:**
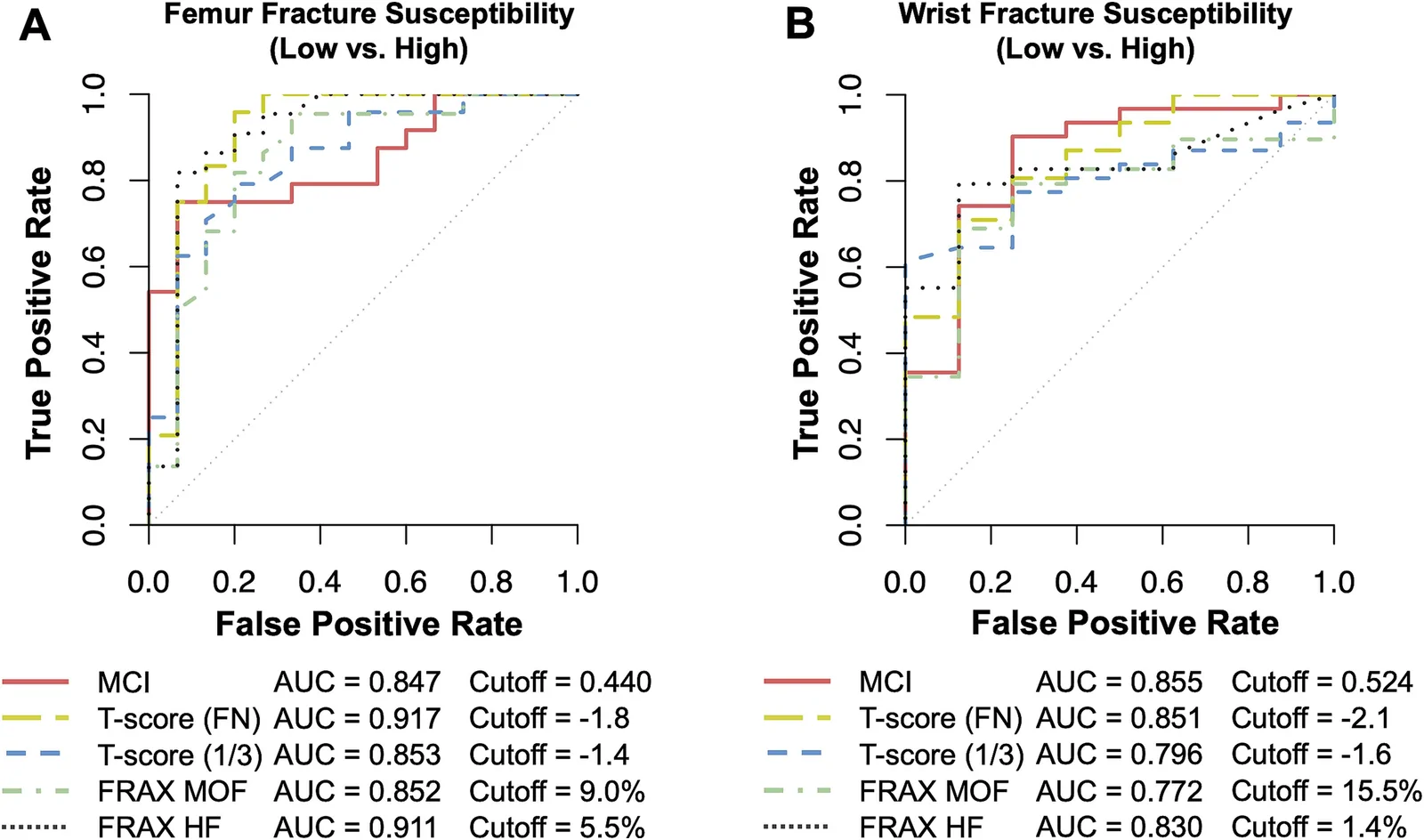
Diagnostic performance of MCI, T-score, and FRAX in classifying low and high femur and wrist fracture susceptibility. (A) ROC analysis evaluating the ability of MCI, FN T-score, 1/3 radius T-score, and FRAX MOF and HF probabilities to classify specimens into low vs high femur fracture susceptibility categories. (B) Corresponding ROC analysis for predictors of low vs high wrist fracture susceptibility classification. AUC values and optimal cutoff points determined using Youden’s index are provided for each predictor, illustrating their relative performance in identifying clinically meaningful fracture susceptibility thresholds. AUC, area under the curve; HF, hip fracture; MCI, metacarpal cortical index; MOF, major osteoporotic fracture; ROC, receiver operating characteristic.

## Discussion

Despite the significant global burden of OP, screening rates remain alarmingly low, underscoring an urgent need for additional, more accessible diagnostic modalities, particularly for populations with limited access to DXA scans. Although the use of MCI to assess OP was first described in 1960,^38^ the limited clinical translation of MCI likely reflects broader historical and translational barriers, including the entrenched reliance on BMD-centric diagnostic frameworks, the lack of standardized MCI measurement protocols, the limited prospective validation, and the uncertainty regarding the systemic relevance of peripheral cortical geometry. To directly test the systemic relevance of peripheral cortical geometry, we evaluated whether MCI reflects bone density and biomechanical fracture susceptibility at clinically important sites. This analysis integrated DXA BMD measurements at both the 1/3 radius and FN, cortical index measurements of the second metacarpal, radius, and femur, and biomechanical assessment of fracture toughness, as a basis for fracture susceptibility classification, by simulating FOOSH and a sideways fall onto the hip. Our findings demonstrated that MCI was highly effective in discriminating between N and OP donors and between OPE and OP donors based on T-score determined from DXA measurements on the 1/3 radius and FN. Comparative analyses of cortical indices across skeletal sites identified MCI as the most effective cortical geometry measure, demonstrating stronger associations with 1/3 radius and FN BMD and biomechanically derived fracture susceptibility than site-specific indices. While FN T-scores and FRAX HF demonstrated the strongest predictions of fracture susceptibility assessment, MCI provided comparable discrimination of low and high biomechanical fracture susceptibility at the femur and wrist, especially when compared to 1/3 radius DXA T-score and FRAX MOF.

All 3 cortical index measurements (MCI, RCI, and FCI) significantly correlated with 1/3 radius T-scores and MCI and FCI with FN T-scores. However, MCI exhibited the strongest correlation coefficient. This likely reflects the greater sensitivity of normalized cortical thickness to age-related thinning in smaller bones with thinner baseline cortices, such as the metacarpal compared to the larger bones of the radius and the femur. In a previous study of the age-related changes in cortical bone at the metacarpal, it was observed that women after age 65 exhibited increased cortical bone loss at the metacarpal of nearly 1% per year, double that compared to the entire study population.^39^ While these changes in cortical bone thinning are not limited to the metacarpals,^40^ differences in mechanical loading and weight-bearing likely attenuate these cortical changes at the radius and femur relative to the metacarpals.

As a predictor of OP progression, MCI effectively distinguished N and OPE donors from OP donors at both the 1/3 radius and FN. Our results are in agreement with findings by Schreiber et al. in which MCI retrospectively distinguished between N and OPE patients (AUC = 0.799) and between N and OP patients (AUC = 0.976) based on hip DXA.^41^ Others have also shown that MCI can be effective in identifying decreased bone density at clinically relevant skeletal sites.^16^^,^^20^^,^^21^ This study extends upon a single skeletal site analysis to illustrate the ability of MCI to predict these classifications at 2 fragility fracture prone sites within a single donor. Our study describes a digital measurement of MCI through FIJI. Recent implementations of medical mobile applications such as the BoneGauge demonstrate the feasibility of integrating this digital measurement into a clinical workflow.^20^ Further steps could also be taken for automated centering and measurement of second metacarpal mid-diaphysis, including the use of machine learning as has been recently described.^42^

Comparative analysis of the peripheral MCI measurement against gold standards for OP diagnosis and fracture risk assessment further emphasized its clinical potential. MCI performed comparably to 1/3 radius T-score and FRAX MOF measurements in the classification of low and high femur and wrist fracture susceptibility. While FN T-score and FRAX HF measurements most effectively classified low and high femur and wrist fracture susceptibility, MCI still exhibited excellent, clinically useful classification.^43^ When examining the cutoffs for the femur and wrist fracture susceptibility based on the FN T-score (cutoff = −1.8 and −2.1, respectively) and the 1/3 radius T-score (cutoff = −1.4 and −1.6, respectively) it is of particular interest that each of these values fall above the OP diagnostic criterion (T-score ≤ −2.5). This result highlights the need for early identification of bone loss and elevated fracture susceptibility during OPE before the OP threshold is met to reduce overall fracture susceptibility. Further, a similar pattern appears when reviewing cutoffs for FRAX. In clinical practice, the threshold for treatment of OP is considered when the 10-year probability of MOF is ≥20% or HF is ≥3%.^44^ FRAX-derived MOF probabilities yielded optimal cutoffs to classify low vs high femoral fracture susceptibility below established clinical treatment thresholds except for FRAX HF prediction of femur fracture susceptibility. This finding indicates that MCI-based fracture susceptibility classification could identify individuals with mechanically vulnerable bone that current FRAX thresholding fails to capture. Given the overall performance of MCI for predicting OP status and biomechanical fracture susceptibility relative to standard DXA-based methods, this approach may be most valuable as a complementary screening tool in resource-limited settings where access to DXA is unavailable. Because MCI can be derived from widely accessible radiographic examination, it may facilitate identification of individuals at risk for OP or fracture who could benefit from further evaluation of treatment. In this intended clinical application, MCI is not proposed as a replacement to DXA where DXA is available or as a secondary imaging examination, but as a complementary screening approach when DXA cannot be obtained.

While this study provides a comprehensive comparison of structural, densitometric, and probabilistic fracture susceptibility metrics on multiple skeletal sites of a female-only cohort to enhance statistical power and clinical relevance of the findings, it has several limitations. The use of cadaveric specimens precludes assessment of neuromuscular contributions to stability and balance, which affect fall risk, and subsequently fracture susceptibility. Fracture susceptibility thresholds were derived from modeled fall energies and represent estimates rather than direct clinical risks. While the direct measurement of bone fracture toughness attempted to simulate fragility fall-related fracture, these tests are limited to a single loading orientation. Falls are complex with many variables across phases of the fall (initiation, descent, impact, and post-impact).^45^ Fragility fracture can be influenced by many factors, including environmental conditions, muscle activity, the properties of the impact surface, and dampening effects from soft tissue, posture, or support on an object. However, by directly measuring consistent simulated fragility fractures, this analysis reduces the number of variables that can affect fracture outcomes in retrospective studies. Another limit of our study is the use of a fracture susceptibility threshold based on a reference female of average height and weight, which may not accurately reflect the susceptibility profiles of individuals at the extremes of body size. Further, limiting the cadaver cohort to women only precludes the direct translation of the study findings to male populations. Future work could focus on developing personalized threshold based on an individual’s height and weight to more accurately assess the susceptibility associated with a fall.

The ground truth classification of the donors as N, OPE, or OP is based on FN DXA BMD measurements of dissected, exposed bone specimens on a preclinical scanner due to logistical constraints. Efforts were made to reduce the potential impacts of ex vivo measurement through conducting measurements in triplicate to capture setup and region of interest variability, standardized thawing and rehydration methods before scans, and analysis setting optimization determined with the scanner manufacturer using “Only Bone” protocols. For 1/3 radius DXA BMD, scanning of the non-dominant forearm is recommended for this measurement, however, this was not available in all cases due to donor availability.^7^ The FN versus 1/3 radius T-score classification results were compared to a large cohort study and illustrated similar levels of agreement (Table S1)^23^ and revealed that the overall concordance between these skeletal site T-scores is ~60%, with major discordance limited to 5%. We present DXA T-scores at both sites throughout this manuscript to characterize the performance of MCI as a remote multi-site screening tool. An additional limitation of this study is the absence of lumbar spine DXA T-scores, which represent a standard clinical site for OP diagnosis and are particularly relevant for vertebral fracture risk assessment. Inclusion of these data points was not possible due to constraints of spine acquisition from the registry company and broader allocation of donor specimens across research projects. Despite this limitation, this study focused on fragility fractures of the wrist and femur as these sites carry among the highest remaining lifetime risk at the age of 45 years and therefore represent clinically relevant sites for evaluating fracture susceptibility.

While donor health histories provided by Anatomy Gifts Registry were comprehensive from all medical records available at the time of death, some details were incomplete. In certain cases, the listed medications reflect hospice or comfort care rather than long-term medical management. Additionally, information on the duration and dosing of medications was not available. Familial history of fracture was not indicated and therefore indicated as a “No” for all FRAX calculations. Information on smoking and alcohol consumption was limited and often quantified descriptively. Interpretations of these descriptors was established to consistently evaluate donors. Although FRAX includes multiple disorders strongly associated with OP for determining secondary OP, we were only able to consistently identify secondary OP in donors with type I diabetes or when it was explicitly documented in the medical history.

Another limitation in the analysis of the cortical indices was the need to account for the relative size of the specimen and the impact that absolute distance measures may have on identifying comparable positions across the radiographs. In particular, FCI measurements were collected 10 cm distal to the mid point of the lesser trochanter as described in Nguyen et al. Given variation in the overall femoral length, a fixed distance from this anatomical landmark could correspond to different relative locations among donors. Future studies could systematically evaluate how the relative distance based on the overall femoral length affect the accuracy in measurement.

Additionally, the biomechanical testing configurations differed between the wrist and femur models. The forearm specimens were tested with soft tissue intact to preserve the complex anatomical relationships between the bones and surrounding soft tissue that contribute to fracture during a FOOSH. In contrast, the femur specimens were tested as isolated samples to minimize variability associated with differences in overlaying soft tissue thickness at the hip. While palmar soft tissue is likely to provide some level of force attenuation, variation in soft tissue thickness of the palm is relatively small compared to that surrounding the hip.^46^ Ultimately, these model selections were chosen to best represent the biomechanics of each anatomical site and should be interpreted in context of these choices.

Finally, the overall sample size of this study was relatively small. This limited cohort may not fully capture the biological variability present in the broader population therefore potentially reducing the generalizability of the findings. In addition, the imbalance in the WHO classification groups based upon the FN T-score was influenced by specimen selection constraints with the cadaveric registry. Donors were selected based on 1/3 radius DXA T-score because FN DXA measurements were not available through the registry unless the full hip was acquired. As a result and due to described levels discordance between DXA measurement sites, the distribution of FN WHO classifications differed from the more balanced 1/3 radius classifications. This limited sample size and class imbalance may have reduced the precision of the diagnostic performance and the robustness of the cutoff thresholds. Given the described study limitations, these reported findings should be interpreted in context of the available DXA measurements, sample size, and biomechanical models evaluated. Future studies including larger, more representative cohorts will be necessary to validate these findings, better capture population variability, and refine clinically relevant threshold values for translation into clinical practice.

We demonstrated the potential of MCI for assessing OP status and evaluating biomechanical fracture susceptibility at 2 common fragility fracture sites: the femur and wrist. Additionally, the study provided further insight into the relationship between cortical indices of the second metacarpal, radius, and femur. These findings suggest that MCI could serve as a practical and opportunistic substitute for DXA, particularly in rural or resource-limited settings where access to DXA is unavailable. Future work should consider prospective clinical studies of MCI’s prediction of fractures using larger, mixed-sex cohorts. Combining cortical indices with clinical risk factors and machine learning approaches may further enhance fracture susceptibility prediction and facilitate wider clinical acceptance.

## Supplementary Material

Supplementary_Data_ziag155

## Data Availability

The data underlying this article cannot be shared publicly due to ethical and privacy considerations related to human-derived specimens. The data will be shared on reasonable request to the corresponding author.
