# Supplementary material for: Cortical indices, DXA, and FRAX as indicators of bone fragility: a comparative human cadaveric study
Source: JBMR Plus. 2026 Sep 14;10(11):ziag155. doi: 10.1093/jbmrpl/ziag155 (PMC13637817; doi:10.1093/jbmrpl/ziag155)
Supplement: Supplementary file 1 [file supplementary_data_ziag155.pdf]

Cortical Indices, DXA, and FRAX as Indicators of Bone Fragility: A Comparative Human Cadaveric Study

Anthony J. Yosick MS, Sophia L. Turbide MD, Andrew J. Berger PhD, Hani A. Awad PhD

Supplementary Data

Supplemental Table 1: Comparison of concordance and discordance levels between 1/3 radius and femoral neck DXA T-score WHO Classification. Concordance refers to agreement between sites. Minor discordance indicates a one-level difference between sites (normal versus osteopenia or osteopenia versus osteoporosis), while major discordance indicates a two-level difference between sites (normal versus osteoporosis). Anticipated percentages are presented in Moayyeri et al. (2005) and measured percentages are collected from this cohort.

	Anticipated Percentage	Measured Percentage
Concordance	58.3%	61.5% (24/39)
Minor Discordance	38.9%	33.3% (13/39)
Major Discordance	2.7%	5.2% (2/39)

Supplemental Table 2: Cohort demographics by 1/3 radius DXA T-score WHO Classification. Demographics are presented as the sample size per classification or as a mean ± standard deviation.

1/3 Radius T-score Classification						
**WHO Classification**	**Donors**	**Ethnicity**	**Age (years)**	**BMI (kg/m^2)**	**BMD (g/cm^2)**	**T-score**
Normal	n = 14	Black: n = 3<br>White: n = 11	59.4 ± 10.1	33.3 ± 8.4	0.699 ± 0.043	0.07 ± 0.71
Osteopenia	n = 10	White: n = 10	63.9 ± 9.0	30.7 ± 7.0	0.598 ± 0.024	-1.58 ± 0.41
Osteoporosis	n = 15	White: n = 15	80.7 ± 9.5	21.7 ± 5.2	0.459 ± 0.056	-3.92 ± 0.93

Supplemental Table 3: Summary of biomechanical parameters computed from force-displacement curves. Values are presented as mean ± standard deviation for each femoral neck WHO classification.

		Femoral Neck WHO Classification		
**Measurement Location**	**Biomechanical Parameter**	**Normal**	**Osteopenia**	**Osteoporosis**
Wrist	Stiffness (N/mm)	263.79 ± 140.78	267.80 ± 160.86	188.18 ± 94.90
	Maximum Force (N)	1882.18 ± 641.80	1797.36 ± 848.03	1252.47 ± 448.31
	Work to Maximum Force (J)	12.70 ± 3.84	11.16 ± 5.36	7.00 ± 3.22
	Fracture Force (N)	1645.82 ± 443.02	1728.84 ± 909.91	1182.14 ± 442.84
	Work to Fracture Force (J)	20.14 ± 10.45	13.05 ± 5.65	9.28 ± 4.95
Femoral Neck	Stiffness (N/mm)	578.94 ± 178.48	548.40 ± 128.29	318.84 ± 133.52
	Maximum Force (N)	4011.06 ± 1056.23	2944.37 ± 597.55	1799.27 ± 491.91
	Work to Maximum Force (J)	35.53 ± 23.33	19.89 ± 12.01	12.28 ± 6.51
	Fracture Force (N)	3573.34 ± 1017.09	2667.49 ± 512.87	1579.20 ± 529.99
	Work to Fracture Force (J)	49.97 ± 16.96	28.87 ± 12.54	19.84 ± 8.57

Supplemental Table 4: Comparison of cortical index between second metacarpal (MCI), radius (RCI), and femur (FCI). R^2, RMSE, and p-value from the regression F-test are listed for each comparison.

Comparison	R^2	RMSE	p-value
MCI vs. RCI	0.173	0.12	0.0055
MCI vs. FCI	0.246	0.11	0.0007
RCI vs. FCI	0.132	0.07	0.0165

Supplemental Table 5: Predictor screening against DXA T-score. Metacarpal cortical index (MCI), radial cortical index (RCI), and femoral cortical index (FCI) variables were regressed against DXA T-scores (femoral neck and 1/3 radius). R^2, RMSE, and p-value from the regression F-test are listed for each comparison.

	**Femoral Neck T-score**			**1/3 Radius T-score**		
**Measurement**	**R^2**	**RMSE**	**p-value**	**R^2**	**RMSE**	**p-value**
MCI	0.487	1.20	<0.0001	0.532	1.29	<0.0001
RCI	0.053	1.63	0.1388	0.146	1.74	0.0115
FCI	0.357	1.34	<0.0001	0.253	1.63	0.0006

Supplemental Table 6: Predictor screening for fracture work. DXA T-score (femoral neck and 1/3 radius), FRAX major osteoporotic and hip fracture risk (MOF and HF; calculated with femoral neck BMD), and metacarpal, radial, and femoral cortical indices (MCI, RCI, and FCI) variables were regressed against fracture work. R^2, RMSE, and p-value from the regression F-test are listed for each comparison.

	**Femur Work to Fracture**			**Wrist Work to Fracture**		
**Measurement**	**R^2**	**RMSE**	**p-value**	**R^2**	**RMSE**	**p-value**
Femoral Neck T-score	0.680	9.13	<0.0001	0.309	6.28	0.0002
1/3 Radius T-score	0.236	14.57	0.0010	0.219	6.85	0.0027
FRAX MOF	0.237	14.73	0.0012	0.198	7.10	0.0058
FRAX HF	0.178	15.28	0.0059	0.178	7.19	0.0094
MCI	0.354	12.98	<0.0001	0.206	6.73	0.0037
RCI	0.088	15.41	0.0660	0.024	7.46	0.3420
FCI	0.117	15.18	0.0334	0.073	7.27	0.0954

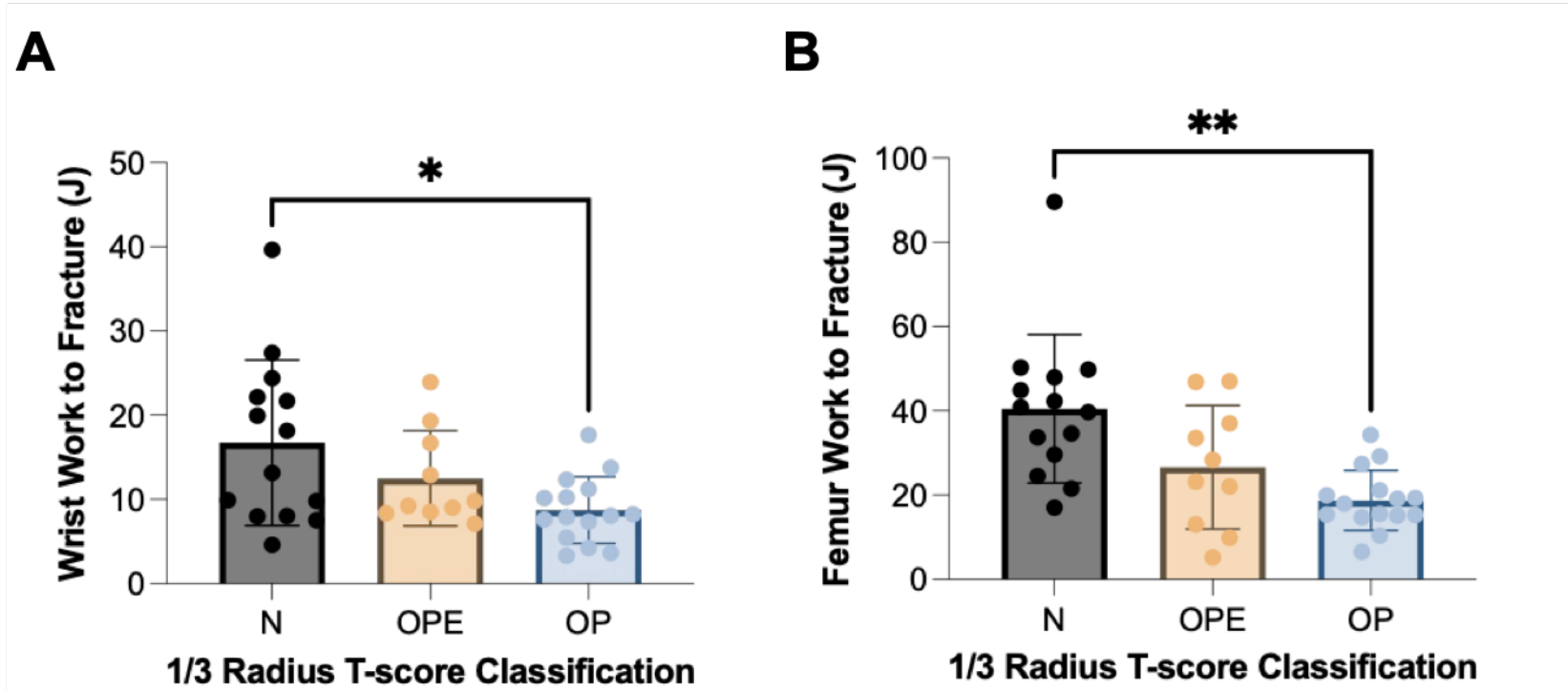

Supplemental Figure 1: Femur and wrist work to fracture values from fall on hip and fall on an outstretched hand (FOOSH) biomechanical models. (A) Femur work to fracture categorized by 1/3 radius DXA T-score. (B) Wrist work to fracture categorized by 1/3 radius DXA T-score. Asterisks indicate significant differences (*p<0.05, **p<0.01).

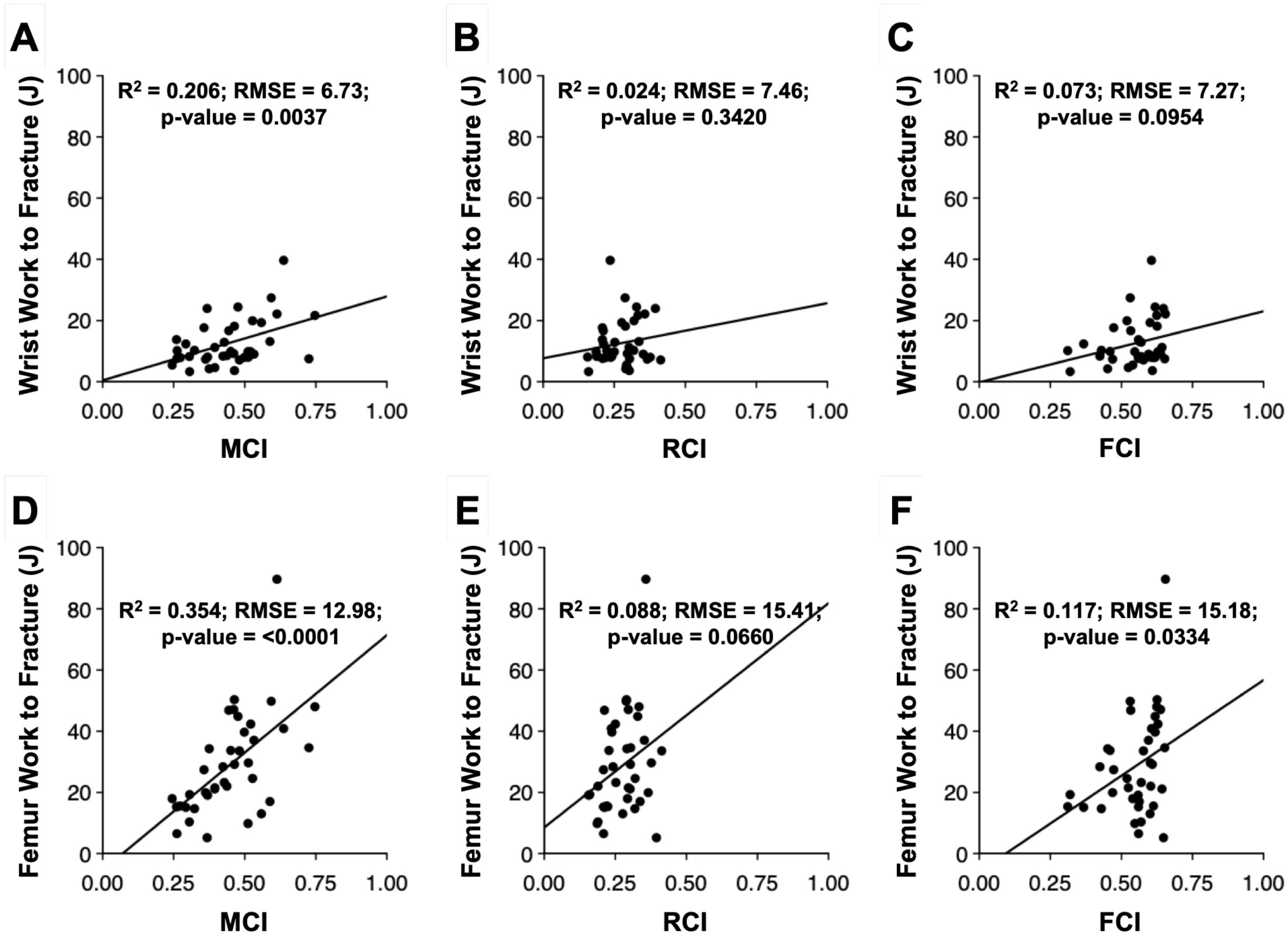

Supplemental Figure 2: Femur and wrist work to fracture values plotted against metacarpal, radial and femoral cortical indices (MCI, RCI, and FCI). (A-C) Wrist work to fracture against MCI, RCI, and FCI. (D-F) Femur work to fracture against MCI, RCI, and FCI.
